# What Next?

**Published:** 1894-10

**Authors:** 


WAAT NEXT?—"Vaccination" against diphtheria as, we suppose the process must be called,—until a name for it is invented—is now an accomplished fact, and the horse, it seems, is to be, like the cow in relation to small-pox, the intermediary and modifyer. See the wonderful wisdom of Providence; nothing is lost, nothing wasted. When tallow began to get scarce and dear, coal oil was discovered; as timber in the older States thinned out, and rails were rails, the barbed wire solved the fence problem. When the electric motor emancipated the horse and the patient mule from the street car service,—what to do with the horse, became a problem, which the French solved by eating him. Behold, science has dedicated the emancipated animal to nobler uses, and he is not yet—*hors du eombat;* he is to be used to knock out diphtheria,—(a kind of *equine*-knocks, eh ?)

A late telegram from Paris says: "Within two months, when more horses have been inoculated, the Pasteur Institute will send out ante-diphtheria serum to the provinces. This serum will also be supplied to druggists in the form of powder."—*Ex.*

Another exchange says: "At a recent session of the State Board of Health of New York, Dr. Cyrus A. Edson gave an account of the theory and practical application of Dr. Koch's last discovery, which he considers an absolute and infallible cure for diphtheria if applied within thirty-six hours after infection. To study and report upon this remedy, Dr. Herman M. Biggs, the bacteriologist of the New York Board of Health, had been sent to Berlin, and has just returned, confirming all the enthusiastic reports concerning the discovery which had made their way to this country.

"Dr. Edson asserted confidently, that if this remedy were placed in the hands of the Health Department, it would save next year the lives of 1500 people in this city."

